# COVID-19 or clinical amyopathic dermatomyositis associated rapidly progressive interstitial lung disease? A case report

**DOI:** 10.1186/s12890-020-01335-z

**Published:** 2020-11-19

**Authors:** Mengke Cao, Shuangshuang Zhang, Dejie Chu, Ming Xiao, Xiaohong Liu, Lingling Yu, Jing Li, Yi Huang, Fang Fang

**Affiliations:** 1 Department of Dermatology, Shanghai Eighth People’s Hospital, 8 Caobao Road, Xuhui District, Shanghai, 200235 China; 2 Department of Dermatology, Jinshan Hospital, Fudan University, Shanghai, China; 3 Department of Dermatology, Shanghai Xuhui District Central Hospital Shanghai, Shanghai, China; 4 Department of Respiratory, Shanghai Eighth People’s Hospital, Shanghai, China; 5 Department of Radiology, Shanghai Eighth People’s Hospital, Shanghai, China

**Keywords:** Clinical amyopathic dermatomyositis, Rapidly progressive interstitial lung disease, COVID-19, Anti-Ro52 antibody, Gottron’s sign, Gottron’s papules

## Abstract

**Background:**

Coronavirus disease 2019 (COVID-19) has reach pandemic proportions globally. For patients with symptoms of fever and cough accompanied by rapid lung damage progression, COVID-19 needs to be distinguished from interstitial lung disease (ILD) attributed to connective tissue disease (CTD), especially dermatomyositis (DM)/clinical amyopathic dermatomyositis (CADM) associated rapidly progressive interstitial lung disease (RP-ILD).

**Case presentation:**

We report a case of a woman observed with fever, cough, and rapid lung damage during the epidemic. The patient had a suspicious epidemiological history, and her chest CT scans showed lung damage similar to that caused by COVID-19, but anti-Ro52 antibody was strongly positive. She was diagnosed with CADM associated RP-ILD and died 1 month later.

**Conclusions:**

During the COVID-19 epidemic, it is critical to carefully assess patients with CTD related ILD, especially RP-ILD associated with CADM. Repeated nucleic acid tests for COVID-19 are necessary to achieve accurate case diagnosis. High-resolution CT (HRCT) of the chest is presently deemed an inefficient technique to distinguishing between COVID-19 and CADM associated RP-ILD. The characteristic rashes of dermatomyositis require careful observation and can often provide diagnostic clues. For patients with CADM, a high titers of anti-Ro52 antibody may be related to the pathogenesis of RP-ILD, suggesting a poor prognosis.

## Background

Currently at the moment of this publications creation there is a worldwide COVID-19 outbreak. For suspected patients with exhibiting symptoms of fever and cough accompanied by rapidly progressive lung damage, COVID-19 needs to be distinguished from ILD caused by CTD, especially CADM associated RP-ILD.

## Case report presentation

A 45-year-old female patient presented with a history of fever, cough, and sputum production for 5 days. From February 6, she had a fever, accompanied by general weakness, chills, muscle aches, as well as a cough. After taking azithromycin orally for 3 days, the symptoms did not improve significantly. CT scans on February 7 showed bilateral, blurred patches, pleural thickening and adhesions. After being admitted into an isolation ward, she was intravenously administered levofloxacin for 3 days but a fever remained. Meanwhile, the results of two consecutive tests for COVID-19 nucleic acid were both negative. She was then transferred to the respiratory single ward on February 11. Upon closer examination of the patient and the medical history, the doctor noted said individual’s developing red, itching rashes on both hands in mid-January 2020. After topical use of miconazole clobetasol cream, the itching symptom reduced while the rashes remained. In this period, she had no muscle aches or myasthenia.

The patient’s previous surgical trauma history included a right lung nodule resection in November 2017 which’s tissue pathology alluded to carcinoma in situ, a thyroid adenoma surgery over 10 years ago and an ovarian cyst surgery in 1999.

At the end of January 2020, she had worked as a radiological technician in a fever outpatient department, but she had no obvious history of close contact with confirmed COVID-19 patients.

**Fig. 1:**
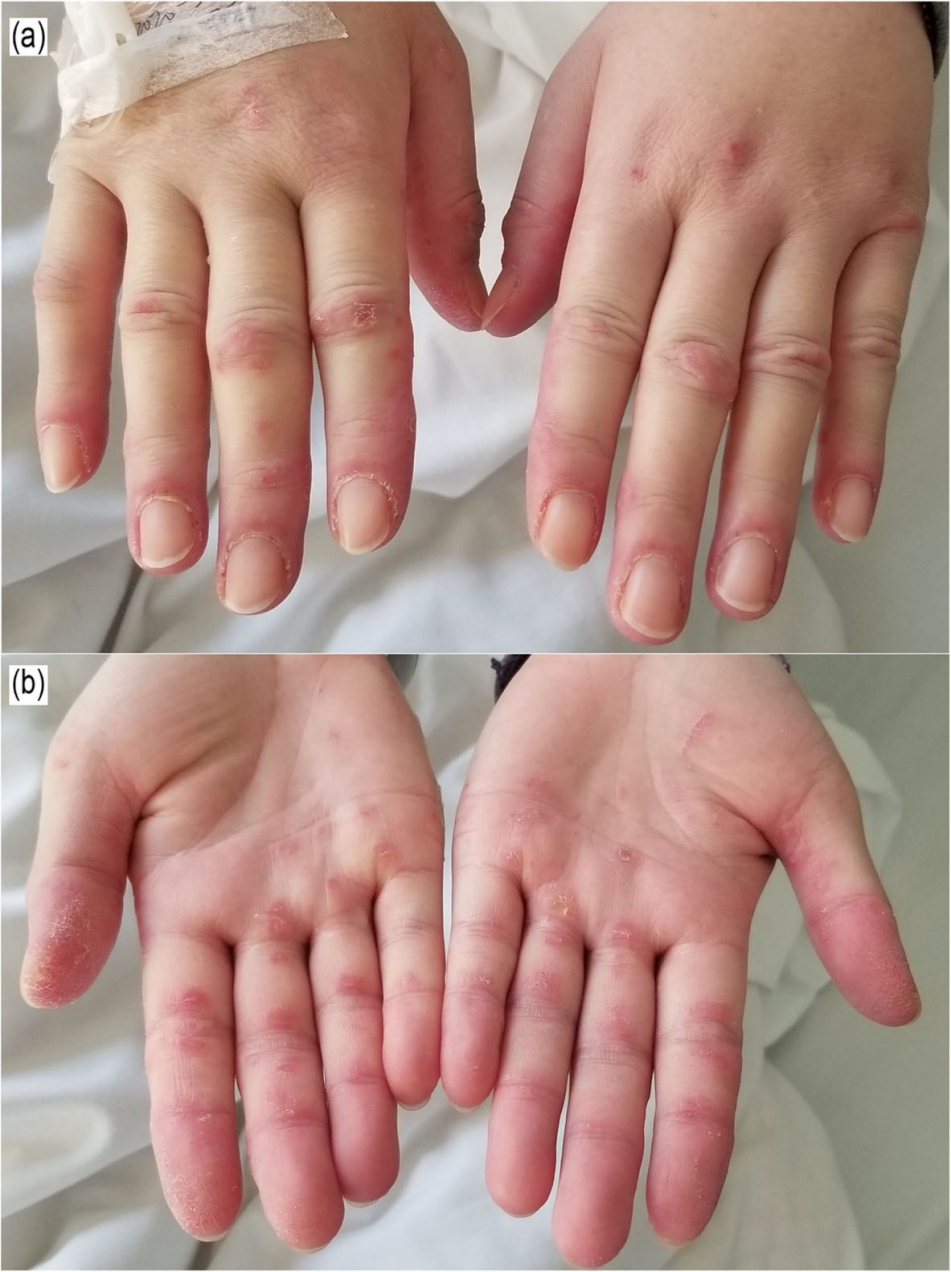
Skin lesions of this case. Erythema and Mung-bean-sized pimples with scales and scab could be seen on the palms, extensor and flexor of knuckles, metacarpophalangeal joints. Periungual folds were diffusely red with angiotelectasis, hyperkeratosis and petechia

A general physical examination revealed smooth breathing with rough bilateral sounds, and wet rales that could be heard at the base of the right lung. Heart and abdominal examinations showed no abnormality. Dermatological examination showed erythema, a condition which mung-bean-sized pimples with scales and scabs could be seen on the palms, extensor and flexor of the knuckles and metacarpophalangeal joints. Periungual folds were diffusely red with angiotelectasis, hyperkeratosis, and petechia (Fig. 1a,b).

Laboratory examinations showed ferritin was 462 ng/ml (Ref. 13~150) and interleukin-6 was 70.05 pg/ml (Ref.0 ~ 7). Creatine kinase and lactate dehydrogenase were higher than normal limits (Table 1). Myositis antibody spectrum examination and autoantibodies showed anti-Ro52 antibody was strongly positive. The examinations including anti-melanoma differentiation-associated gene 5 (anti-MDA5) antibody, anti-Jo1 antibody, anti-Scl70 antibody, antinuclear antibody, anti-double-stranded DNA antibody, anti-SSA/SSB antibodies, anti-Sm antibody and antineutrophil cytoplasmic antibody were all negative. Pathogen examination of respiratory system produced no significant results.

**Table 1:** Lab results during the treatment

	12/2/2020	17/2/2020	19/3/2020	Reference range
Red blood cells (×10^12^/L)	4.07	3.68	3.98	3.8-5.1
White blood cells (10^9^/L)	4.82	7.60	16.7	3.5-9.5
Neutrophil (%)	67	83.50	93.9	40-75
Lymphocytes (%)	27.2	11.2	3.7	20-50
Absolute neutrophil count (10^9^/L)	3.23	6.35	15.7	1.8-6.3
Absolute lymphocytes count (10^9^/L)	1.31	0.85	0.6	1.1-3.2
Hemoglobin (g/L)	121	109	118	115-150
Platelet count (10^9^/L)	170	167	274	125-350
Hypersensitive C-reactive protein (mg/L)	2.31	24.19	25.2	0-10
Erythrocyte sedimentation rate (mm/h)	38	44	1.68	0-15
Creatine kinase (U/L)	152	244	48	30-135
Lactate dehydrogenase (U/L)	324	368	1037	120-250
D-dimer (mg/L)	2.77	2.84	1.68	0-1

After in-patient admission, above mentioned subject was given a combination of anti-infective therapy for 1 week, but afterwards she still exhibited hyperthermia. On February 17, CT imaging revealed progressive changes and the oxygenation index showed a sharp decline. After a multidisciplinary consultation, she was immediately transferred to the ICU quarantine ward. After 3 days of active treatment, she still had a 39.5 °C fever, and her oxygenation index continued to deteriorate. At this time, the third nucleic acid test for COVID-19 was administered indicated a negative result. Comprehensively considering the patient’s clinical manifestations and auxiliary examination results, she was diagnosed with CADM associated RP-ILD. The patient was immediately given non-invasive ventilator-assisted breathing, methylprednisolone (up to 120 mg/day), gamma globulin (0.4 g/kg/d), and cyclophosphamide. The body temperature began to dissipate and the condition exhibited improvement. However, after glucocotrticoids reduction the lung lesions continued to progressed dramatically over a few days and culminating in said patient’s mortality a month later.

## Discussion and conclusions

Patients in China presenting symptoms such as a fever and/or cough during this COVID-19 outbreak should be treated with caution. Although the patient had no clear epidemiological history of COVID-19 exposure, she was engaged in radiograph work as a technician in the fever clinic for 1–2 weeks before her fever manifested. During this period, a confirmed COVID-19 patient was examined there but did not have direct contact with our patient. Because little was known about COVID-19 at that time and the nucleic acid test can produce false-negatives, COVID-19 initially remained as a potential diagnosis despite negative findings from two consecutive tests.

The onset of DM in this patient was a brief period under 15 days from the rash’s appearence on her hands to the development of fever and cough. In addition, the patient had no obvious symptoms of muscle soreness and weakness, typical purplish red edematous patch around the palpebra superior, and eye sockets except for Gottron’s sign / Gottron’s papules on her hands. Gottron’s sign/ Gottron’s papules is a red flat/raised papular rash on the extension side of the finger joint of both hands, which is a significant diagnostic sign of DM. It is considered that the patients with typical DM as the main manifestation, no myositis symptoms for at least 6 months or mild abnormalities in laboratory tests are collectively referred to as CADM at present [1]. CADM patients often have skin manifestations of DM in the early stage of the disease, over a span varying from weeks or months, RP-ILD suddenly appears, which is potentially accompanied by fever, dyspnea, and rapid progress to respiratory failure. At that time, the imaging manifestations of both lungs of CADM patients are diffuse exudation with ground glass opacity and other interstitial changes, with a 6-month survival rate of 54.5% [2], especially in East Asia [3–5]. Combined with Gottron’s sign / Gottron’s papules, slightly elevated creatine kinase levels and strongly positive anti-Ro52 antibody, multiple fuzzy patches and consolidation of both lungs with pleural effusion, the patient reported was diagnosed as CADM associated RP-ILD. Other classic cutaneous signs of DM / CADM include poikiloderma, periorbital heliotrope eruption, shawl sign and periungual telangiectasias. Therefore, understanding the characteristic rashes of DM is crucial for the diagnosis of DM /CADM.

**Fig. 2:**
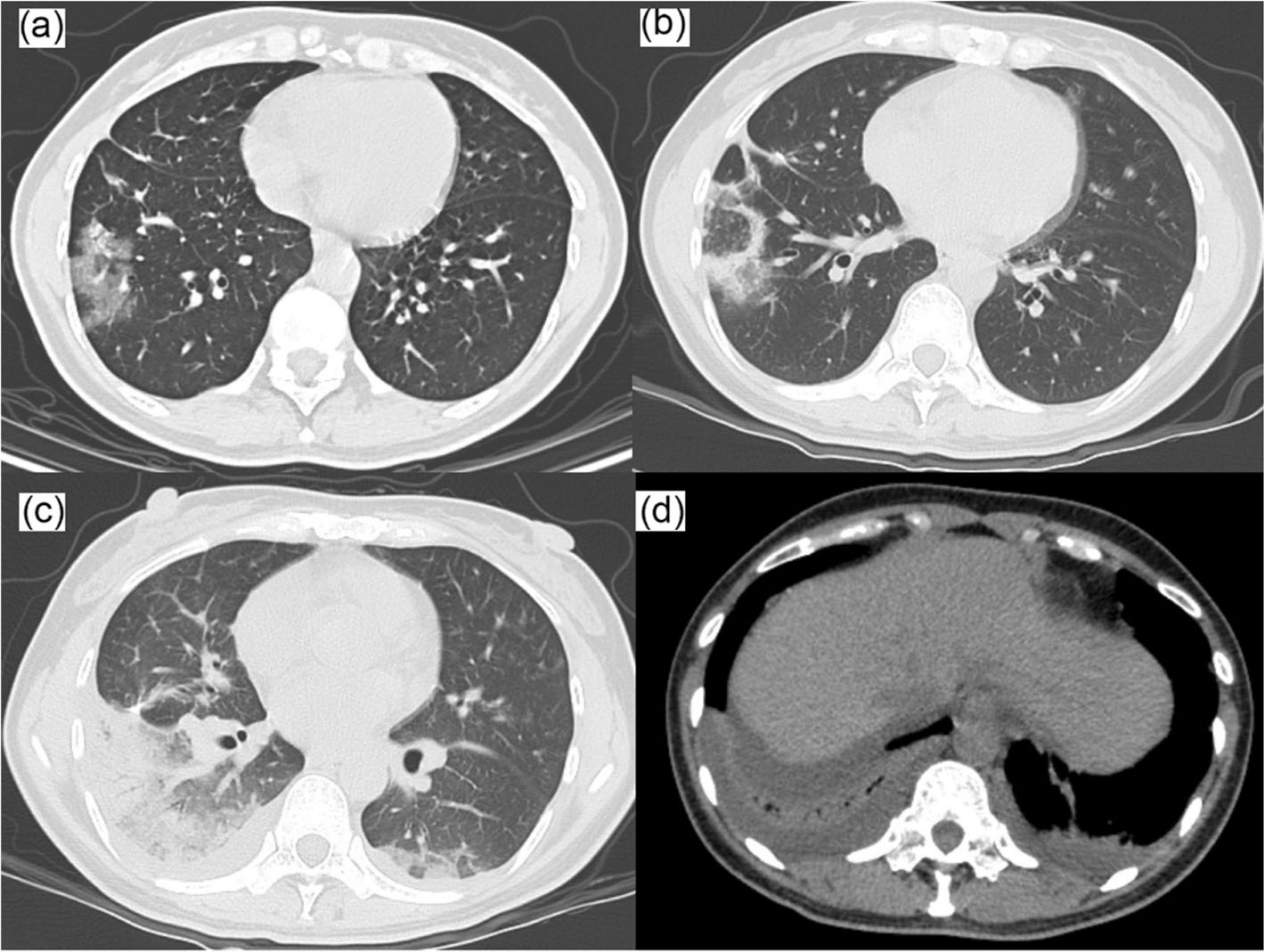
Chest CT scans. **a** Bilateral, blurred patches, pleural thickening and adhesions can be seen on February 7, 2020. **b** progressive changes with more patchy shadows on February 11, 2020. **c**, **d** Diffuse infiltrates with ground-glass opacities in the right lower lobes and bilateral Pleural effusions on February 17, 2020

The CT findings of CADM associated RP-ILD are difficult to distinguish from those of COVID-19. The CT scans of this patient showed ground-glass opacities and patchy shadows in the bottom of the right lung (Fig. 2a,b); however, these developed into multiple blurred patches (Fig. 2c) and significant pleural effusions (Fig. 2d) after several days. These are very similar to COVID-19: the most common CT manifestations of COVID-19 were ground glass opacity, consolidation, and reticular pattern, etc., but the incidence of pleural effusion was reported to be 1–8%, and the occurrence of pleural effusion suggested that the prognosis of patients with COVID-19 was terrible [6]. Therefore, using HRCT is difficult to distinguish COVID-19 and CADM associated RP-ILD.

Dermatomyositis (DM)/polymyositis (PM) is a disease spectrum and CADM is a related unique variation. Studies have shown that some specific antibodies are related to the prognosis of CADM. The adverse prognosis of CADM associated RP-ILD is often considered to be related to anti-MDA5 antibody [7–9]. The patient had a poor prognosis, however, the result of anti-MDA5 antibody was negative. This led us to consider the role of high titers of anti-Ro52 antibody. Anti-Ro52 antibody has been discovered and received greater study in recent years. It has been reported that high titers of anti-Ro52 antibody is associated with the poor prognosis of myositis associated ILD [10–12]. For example, studies have suggested that the most common myositis-related antibody in adult idiopathic inflammatory myopathy is anti-Ro52 antibody [13]. The high titers of anti-Ro52 antibody is more relevant to severe ILD [10], whereas anti-Jo1 antibody is increasingly associated with the occurrences of myositis and joint damage [11]. Li et al. had analyzed 130 cases of DM/PM patients and found that DM/ PM-ILD patients were more likely to develop anti-Ro52 antibody than those without ILD in DM/PM [14]; another study by the Massachusetts General Hospital found that anti-Ro52 antibody was helpful in distinguishing non classified ILD, especially it could become a serological marker of interstitial pneumonia with autoimmune features (IPAF) [15]. Hudson et al. [16, 17] found that anti-Ro52 antibody was closely related to systemic sclerosis (SSc) associated ILD in 963 SSc patients in Canada, and the mortality risk of those patients with positive anti-Ro52 antibody was significantly higher than that of negative patients. Therefore, the high titers of anti-Ro52 antibody may be related to the poor prognosis of CADM associated RP-ILD. At least, it can be deduced that the high titers of anti-Ro52 antibody may be related to the poor prognosis of autoimmune ILD.

This case serves as a reminder that during the COVID-19 epidemic, it is critical to carefully assess patients with CTD related ILD, especially RP-ILD associated with CADM. Repeated nucleic acid tests for COVID-19 are necessary. The characteristic rashes of dermatomyositis require careful observation and can often provide diagnostic clues. It’s difficult to distinguish between COVID-19 and CADM associated RP-ILD from HRCT. For patients with CADM, a high titers of anti-Ro52 antibody may be related to the pathogenesis of RP-ILD, suggesting a poor prognosis.

## Data Availability

All data generated or analysed during this study are included in this published article.

## Abbreviations

COVID-19: Coronavirus disease 2019
ILD: Interstitial lung disease
CTD: Connective tissue disease
RP-ILD: Rapidly progressive interstitial lung disease
DM: Dermatomyositis
PM: Polymyositis
CADM: Clinical amyopathic dermatomyositis
IPAF: Interstitial pneumonia with autoimmune features
SSc: Systemic sclerosis
Anti-MDA5 antibody: Anti-melanoma differentiation-associated gene 5 antibody
